# Assessing the environmental impacts of agrifood production

**DOI:** 10.1007/s10098-021-02153-5

**Published:** 2021-07-10

**Authors:** Ittisak Jirapornvaree, Tawadchai Suppadit, Vikas Kumar

**Affiliations:** 1 Graduate School of Environmental Development Administration, National Institute of Development Administration, Bangkok, 10240 Thailand; 2 Faculty of Accounting, Ton Duc Thang University, Ho Chi Minh City, Vietnam; 3 Bristol Business School, University of the West of England, Bristol, BS16 1QY UK

**Keywords:** Environmental management, Life cycle assessment, Organic agricultural system, Jasmine rice production, Sustainable agricultural development, Agripolicy

## Abstract

Agriculture, forestry, and other land use approximately contribute to 24 percent of 2010 global greenhouse gas (GHGs) emissions. This study, therefore, focuses on assessing the environmental impacts of jasmine rice production. The life cycle assessment method is used to find the hotspots that are of high environmental impacts. Face-to-face interviewing was conducted with 49 rice producers engaged in chemical, organic, and good agricultural practices in farming activities. The results show that most of the emissions were caused during the post-harvest management stage. Following eco-efficiency, organic jasmine rice production offers a reduction in the consumption of resources, reduced impact on the environment, and increased product value. Furthermore, our study shows that crop residue is a key to increase rice yields and decrease GHGs emissions. Our findings thus add to the limited literature on organic jasmine rice production and propose a recommendation for policymakers to promote sustainable agricultural practices to reduce the environmental impact.

**Graphic abstract:**

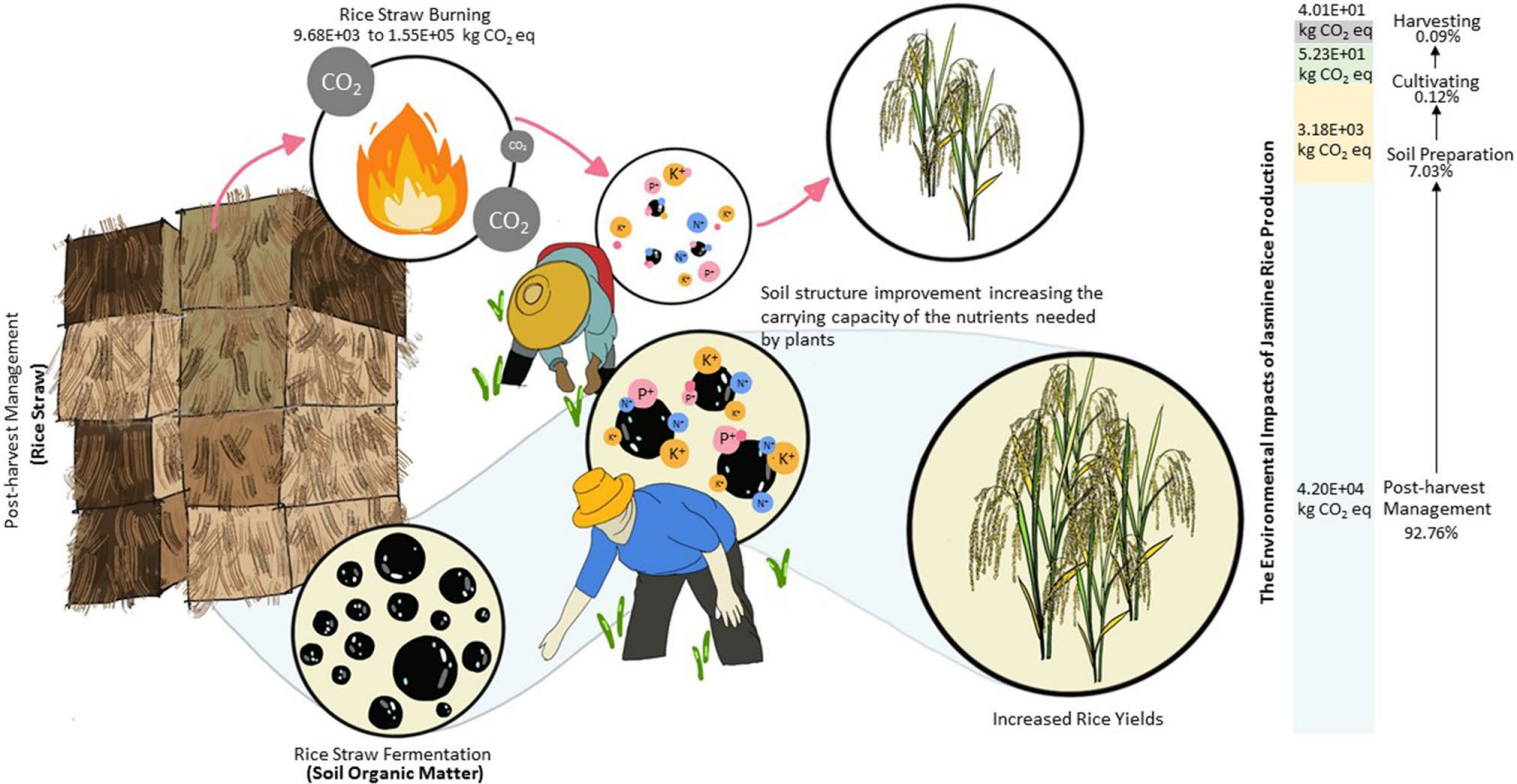

## Introduction

World economic development in the past 30 years has compelled many countries to review the results of their past operations. In 2015, Sustainable Development Goals (SDGs) were developed by the United Nations to guide for improved products to meet their own needs. Most countries around the world are therefore determined to develop their country to be stable and sustainable. Pingali (2012) showed that the green revolution has affected agricultural productivity improvement. They found high poverty reduction with agricultural productivity growth. In Asia, every one percent productivity growth contributes to the reduction in poor people by 0.48 percent (Thirtle et al. 2003). On the other hand, this affects the environment negatively. Lobell et al. (2008) stated that climate risk poses significant challenges to several crops in South Asia and Southern Africa region affecting food security which is also echoed by Masutomi et al. (2009). In this line, Brown and Funk (2008) further reported that in recent years rice yields were reduced by increasing temperatures and declining precipitation. These changes are affecting global food security. Food security improvement should be concentrated on the quality and quantity of agricultural production to respond to their food needs (Food and Agriculture Organization 2018a). The ongoing COVID-19 pandemic further adds to these woes affecting the staple food production that consists of wheat, coarse grains, and rice (FAO 2020).

Rice is the staple food crop for most Asians. Asia produces some 90 percent of the world’s total supply of rice (Chapman et al. 2019). The total proportion of land for rice cultivation, as compared with total arable land, is highest in Vietnam, Bangladesh, and Sri Lanka. In terms of white rice, India, Thailand, and Vietnam are notable rice exporters. Meanwhile, jasmine rice that is produced in Thailand has a 60 percent of the world market share whereas Vietnam and Cambodia hold 23 and 8 percent market share, respectively (Office of the Permanent Secretary Ministry of Commerce 2018). In 2016, the Food and Agriculture Organization (2018b) reported that agriculture used more than 50 percent of the total land in Asia. Moreover, the world total of chemical or mineral fertilizers use was 110 Mt nitrogen (N), 49 Mt phosphate (P_2_O_5_), and 39 Mt potassium (K) in the agriculture sector. In Asia, China, India, Pakistan, Indonesia, Thailand, and Vietnam are the major fertilizer users. While increases in the use of fertilizers like nitrogen have had a positive impact on agricultural production, they have also had notable negative effects on human and environmental health and thus reduced usage of fertilizers is good for the environment (Pardey et al. 2008; Avagyan 2008). Cereals make up the bigger part of crop production. Among the top five items produced in 2016, sugar cane was equal to 1,890,662 thousand tons. Meanwhile, rice and paddy were the fourth items equal to 740,961 thousand tons. Indonesia, China, India, Malaysia, and Brazil alone covered nearly 40 percent of the permanent crops (Thailand is 11th of these). Currently, there is increasing consumption of rice and among many factors, a growing world population and agricultural growth are the key factors contributing to this increase.

As it is evident from the discussions, there are limited studies that have attempted to focus on exploring sustainable agricultural production methods while also focusing on improving the yield. For example, a study by Bacenetti et al. (2016) attempted to assess the environmental profile of organic rice cultivation in a farm located in Pavia District (Lombardy, Italy) using the LCA method. Moreover, there are limited studies that have either attempted to explore the environmental impact of rice or production efficiency in the Thai context (Yodkhum et al. 2018; Rahman et al. 2009). Given that rice consumption has increased in recent years there is a need to explore more sustainable methods. Therefore, this study aims to identify the hotspots and investigate the environmental impacts of jasmine rice production, with the hope of increasing the yield as well as decreasing the production costs and GHG emission impacts. The purpose is to identify an alternative agricultural approach that promotes sustainability and security in the food production system. It is also aligned with current trends in ecologically sustainable production and support Wollenberg et al. (2016) who suggested that we should reduce the emissions from agriculture to meet the 2 °C targets following the aim of the 2015 Paris Agreement of the United Nations Framework Convention on Climate Change (UNFCCC). Rest of the paper is structured as follows; “Literature review” section describes the methodology adopted in this study and study area. “Methodology” section presents the findings and discussions. Finally, “Conclusions” section concludes this study by highlighting the implications, limitations, and future research directions.

## Literature review

### Rice production in Thailand

Rice cultivation areas in Thailand is approximately 8.970 million hectares (Rice Department 2019), and rice production is 25.18 million tons (Office of Agricultural Economics 2019). Most Thai cultivated areas are based on rainfed which is approximately 85 percent of the total rice yields of Thailand. In 2019 (March 01–September 30, 2019), the northeastern region was the area where most of the rice cultivation took place and cultivating approximately 5.638 million hectares (63 percent of Thailand rice cultivated) (Rice Department 2019). Table 1 shows the rice cultivation in different regions of Thailand and for different types. Thung Kula Rong Hai (TKRH) region in northeastern Thailand was selected for this study as this area is known for jasmine rice production, particularly jasmine rice 105 for which yields is approximately 567 kg per rai (Rice Department 2020).

**Table 1:** Rice cultivated areas in Thailand 2019 separated by type of rice. *Source* Rice Department (2019)

Region	Cultivated areas	Type of rice			
		Jasmine^a^	Thai jasmine^b^	Thai pathumthani	Others
North	2.061	0.463	0.069	0.035	1.494
Northeastern	5.638	3.575	0.000	0.003	2.060
Central	1.221	0.201	0.180	0.082	0.758
Southern	0.050	0.002	0.014	0.003	0.031
Total	8.970	4.241	0.263	0.123	4.343

Unit: million hectare

*a* = Jasmine rice 105 or 115 was cultivated in TKRH

*b* = Thai jasmine rice was cultivated in Thailand

Many factors challenge the development of stable and sustainable agriculture. Food security is at risk from climate change as stated earlier (FAO 2020; Schmitz 2017). Drought and saltwater intrusion affected the cultivation of vegetables in Bangladesh, the Philippines, and Vietnam (Arunrat et al. 2018; FAO 2020). In 2019, the rice yields in Thailand and Indonesia fell due to delayed plantings from the precipitation (Prabnakorn et al. 2018). Topography and the soil characteristics have a limited response to the use of fertilizers. Hence, the addition of chemical fertilizers in soil did not increase the yields but rather increased the production costs.

### Environmental impact of rice production

Agriculture GHG emissions contributed about 5 billion metric tons of CO_2_eq to the atmosphere each year during the period 2005–16 (Food and Agriculture Organization 2018b). Food is approximately contributing to 26 percent of global GHG emissions (Poore and Nemecek 2018), and Asia is the region with the largest share of emissions. Thailand is the 20th in GHG emissions in agriculture. One of the increasing agricultural production problems is that the world agricultural system overproduces grains, fats, and sugars. The world production will increase the total land used for agriculture, but it is not focused on the yield and will contribute to increased emissions (Kc et al. 2018). Hence, there is a need to explore more sustainable agricultural production methods that would be better for the environment while also resulting in better yield. Life cycle assessment is widely used for achieving sustainable agricultural goals (Habibi et al. 2019; Islam et al. 2017). He et al. (2018), Mungkung et al. (2019), and Yodkhum et al. (2017) opted for LCA method to find the hotspot where there are high GHGs emission impacts from agricultural production. At the same time, Winkler et al. (2016) selected this method to study the livestock sectors in order to improve production. LCA has helped to decrease the emission and costs of agricultural production. The method was chosen to compare and confirm the alternative approach that has the potential to reduce GHG from agricultural sectors (Llorach-Massana et al. 2017; Zhang et al. 2019). This also helps to increase understanding of the relationship between human activities and environmental impact which is useful for sustainable development. The result from LCA is simplified to inform the decision to implement in field or policymaking (Ruviaro et al. 2012).

To sum up, LCA technique is part of tool which understand insight in agrifood production chains, consistent with Aertsens et al. 2009 reported that LCA is tool to address questions on the environmental impact of agrifood production. These can identify the hotpots and the comparison of products and process with the same function. These are also used to make a decision to select approach or material in various agrifood productions.

## Methodology

This study adopts a qualitative approach. The findings are based on the face-to-face interviews of 49 farmers engaged in jasmine rice production (jasmine rice 105/115) in Thung Kula Rong Hai (TKRH), a northeastern region of Thailand. A purposive and snowball sampling method was used to identify the participants. These participants were separated in three groups as follows; 31 chemical agriculture farmers; 4 good agricultural practices (GAP) farmers; and 14 organic agriculture farmers that were selected using Codex Guidelines on the production, processing, and marketing of organically produced foods such as Organic Thailand, ACT Organic Standards by Organic Agriculture Certification Thailand and IFOAM by International Federation of Organic Agriculture Movements. These must have been cultivating for more than five years as the standard for further comparison.

**Fig. 1:**
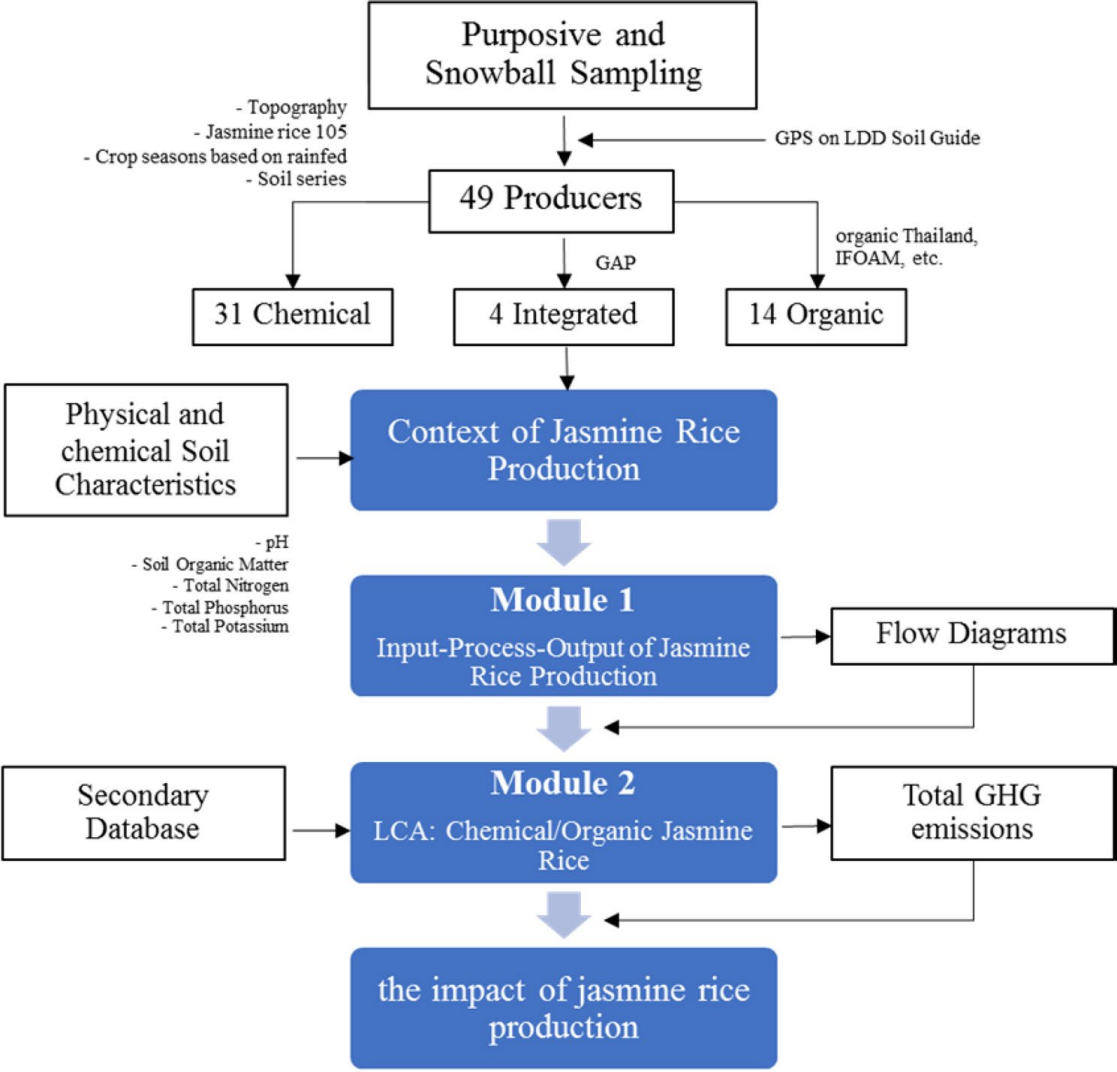
Research process flow of this study

In terms of the context of physical and chemical jasmine rice production, soil pH, Walkley Black modified acid–dichromate digestion, total Kjeldahl nitrogen (TKN), total phosphorus (using the colorimetric method), and exchangeable potassium were measured. Input–process–output was chosen to study the jasmine rice production activities flow. Environmental impacts between the chemical, GAP, and organic jasmine rice production were assessed using the LCA method. A four-step process followed in this study included goal definition and scoping; inventory analysis; impact assessment; and interpretation. CML (baseline) [v4.4, January 2015] (e.g., global warming (GWP100a), acidification, and eutrophication) was used to explain data. Therefore, the research process flow of this study is shown in Fig. 1.

### Study area

Thung Kula Rong Hai (TKRH) region consists of five provinces and thirteen districts as shown in Table 2. This area is protected by Geographical Indication by the Council Regulation (EC) No. 510/2006 since 2006 (Rice Department 2006). This area is approximately 320,000 hectares (2 million rai) and annual rainfall in the area is approximately 1100–1400 mm, especially during the rainy season (March–November). The land consists of sandy and salty soil that cannot absorb water. Chemical characteristics are low soil fertility and soil organic matter.

**Table 2:** Soil characteristics and rice yield in TKRH, Thailand

Provinces	Districts	Soil characteristics	Rice yields
Roi- Et	Kaset Wisai	Soil fertility was low, pH was strong acid (4.3), phosphorus available was moderate (1–10 ppm), and potassium exchanged was low (1–10 ppm) (Rice Department 2013)	89.60 kg/ha
	Suwannaphum		
	Nong Hi		
	Phon Sai		
Maha Sarakham	Phayakkhaphum Phisai	Soil fertility was low, pH was strong acid (4–5), OM was low (lower than 1%), phosphorus available was high, and potassium exchanged was low (Office of Permanent Secretary Ministry of Industry 2017)	69.92 kg/ha
	Pathum Rat		
Surin	Chumphon Buri	Soil fertility was low, pH was strong acid (3.5–5), OM was low (lower than 1%), phosphorus available was moderate (10 ppm), and potassium exchanged was low (10–50 ppm) (Kannikha Nakhang et al. 2007a, b)	52.16–89.60 kg/ha
	Tha Tum		
Si Saket	Rasi Salai	Soil fertility was low, pH was strong acid (4.5), OM was low (0.5%), phosphorus available was moderate (11 ppm), and potassium exchanged was low (24 ppm) (Kannika Nakhang et al., 2007a, b)	72.00–87.20 kg/ha
	Yang Chum Noi		
Yasothon	Maha Chana Chai	Soil fertility was low, pH was strong acid (2.68 – 5.70), OM was low (0.192 – 0.947%), phosphorus available was low to high (1.24 – 25.80 ppm), and potassium exchanged was low (5.11 – 49.04 ppm) (Ubon Ratchathani Rice Research Center 2009)	56.00–72.00 kg/ha
	Kho Wang	Soil fertility was low, pH was strong acid (3.05–4.66), OM was low (0.401–0.754%), phosphorus available was low to moderate (1.87–6.41 ppm), and potassium exchanged was low (8.42–35.25 ppm) (Ubon Ratchathani Rice Research Center 2009)	

Most of the soil characteristics in the northeastern region are the Altisoll and Sodick soils which are saline. These findings are consistent with those of Cha-um and Kirdmanee (2011); Cha-Um et al. (2009) who reported that these soils are not suitable for the production of general rice. On the other hand, jasmine rice is more resistant to the adverse environment than general rice (Yoshida and Parao 1976). Due to the unsuitable conditions of these soils such as salinity, dehydration, low nitrogen and phosphorus, and high sodium, it promotes stress condition on jasmine rice resulting in the increased aroma of rice (Dubey and Singh 1999; Wanichananan et al. 2003). Hence, the quality of rice in this region is considered good quality.

Table 2 shows that soil pH in TKRH is very strong to slightly acidic (3.50 to 5.70). The soil organic matter (OM) is low and total nitrogen, phosphorus, and potassium remained at low to moderate levels. To sum up, soil quality in TKRH is suitable for jasmine rice production but it is not suitable for increasing the rice yields.

### Life cycle assessment (LCA) study

Life cycle assessment (LCA) is necessary for decreased risk and supports the growth of rice production. It concentrates on elimination or reduction in products that are not needed (Schaltegger 2014). This is an environmental management tool that informs decision makers other decision criteria such as cost and performance that should be considered to make a well-balanced decision (Curran 2008). The analysis was performed using openLCA software, an open assess program.

#### Goal and scope definition

**Fig. 2:**
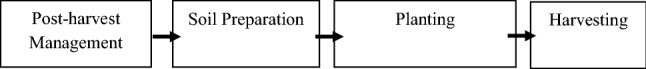
System boundary of this study

The goal of this analysis is to determine the environmental impacts of the conventional, GAP, and organic jasmine rice production in TKRH, Thailand. The system boundary covers from Cradle-to-Gate (see Fig. 2) as shown in data interpretation.

#### Life cycle inventory (LCI)

**Fig. 3:**
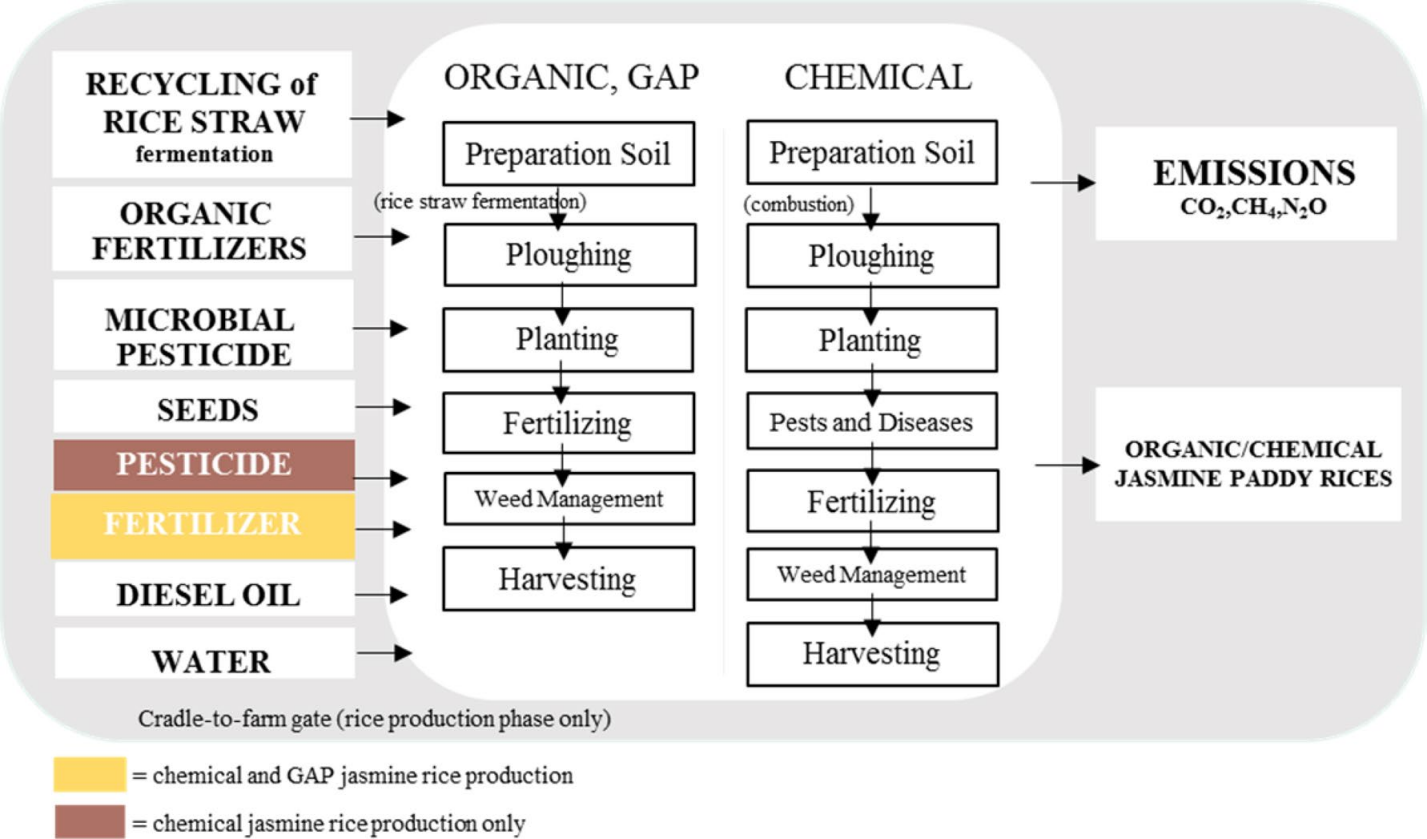
Conventional and organic jasmine rice production flows

Emission factors of input data consisting of all the input materials, energy consumption, and all the related and output data were collected from the life cycle inventory database by Thailand Greenhouse Gas Management Organization (Public Organization) and Thai National Life Cycle Inventory (see Table 3). Before the data collection, the inventories were prepared based on the process flow diagram as shown in Fig. 3. LCI data were collected from the rice production practices of three rice patterns from forty-nine farmers through a face-to-face interview. All data of the inventories, including all the inputs during the whole production process, were analyzed in relation to the functional unit (1 kg of rice produced) by setting assumptions and calculation of CO_2_ equivalent from chemical, organic, and GAP jasmine rice production.

**Table 3:** Emission factors of parameter and input

Parameter/Inputs	Units	Data source	Emission factor		
			Chemical	GAP	Organic
Average yield	kg/ha	Interviewing	368.72	392.46	520.09
Crop period		Interviewing	1^st^ (May–November)		
Rain water			Rainfed		
Land	ha	Interviewing	0.8 – 1.6 1.6 – 7.2 > 7.2		
Jasmine rice seed	kg	TGO	0.2500		
Green manure seed	kg	TGO	–	0.6999	
Organic fertilizer production	kg	TGO	–	0.1638	
Cattle manure	kg	TGO	–	0.1097	
Bio fermentation production	l	TGO	–	0.1638	
Chemical fertilizer 21%N	kg	TGO	3.3036		–
Chemical fertilizer 46%P_2_O_5_	kg	TGO	1.5716		–
Chemical fertilizer 60%K_2_O	kg	TGO	0.4974		–
Herbicides (Glyphosate)	l	TGO	16.0000		–
Emission from rice straw burning	kg	IPCC*	1.5150		–
Diesel oil production	l	TGO	0.3522		
Diesel oil combustion	l	TGO	2.7446		
transportation by 7-ton truck 100% loading	kg km	TGO	0.1411		

*TGO* = Thailand Greenhouse Gas Management Organization (Public organization), 2020

* = Based on IPCC default value (Burning of dry matter–agriculture residues)

#### Life cycle inventory assessment (LCIA)

The life cycle GHG emissions were calculated by adopting Product Category Rules of rice product based on TKRH, Thailand. The direct methane emissions from the rice cultivated are based on country-specific emission factors (Tier-2 methodology), and nitrous oxide emissions from fertilizer application are based on Tier-1 methodology. Moreover, this study concentrated on air and water pollutions as follows:

##### Global warming potential (GWP)100 (100-year time horizon)

The emissions of jasmine rice production were made by human activities. The GHG emissions such as CO_2_, CH_4,_ and N_2_O are applied (IPCC 2006). It is expressed in terms of mass (e.g., kgCO_2_ equivalents), which have a GWP of 1, 25, and 298, respectively.

##### Acidification potential

Sulfur dioxide (SO_2_), ammonia (NH_3_), and nitrogen oxides (NOx) were focused at this point. They are emitted by burning rice straw and causes “acid rain.” It is expressed in kg-SO_2_ equivalents.

##### Eutrophication potential

The emissions of jasmine rice production were made by the runoff of synthetic fertilizers from agricultural land. nitrogen (N) and phosphorus (P) are used in this analysis. It is expressed in phosphate (PO_4_) equivalents.

#### Data interpretation

Finally, the results of LCI and LCIA were analyzed. It was done based on the goal and scope. Overall, the impact of jasmine rice production was compared between the organic, conventional, and GAP process. The impact on the environment by rice productions was identified. Finally, the recommendations are provided based on the negative impacts on the environment.

## Results and discussion

### The context of jasmine rice production

The findings of this study are based on interviews with 49 farmers engaged in jasmine rice production. This study found that chemical, organic, and good agricultural practices of jasmine rice production are the main approaches in Thung Kula Rong Hai (TKRH). Rice cultivation in this area is rainfed (May to December) as opposed to the central region where the production cycle of rice is two to four times per year. Rice production flow is simple cultivation including post-harvest management, soil preparation (1 month), planting (4 months), and harvesting. Pesticide and herbicide are relatively used in small quantities in these regions as producers aim to make fallow soil after the harvesting for four to five months to maintain soil and eliminate the weed. Jasmine rice 105 and 115 were chosen for cultivation in these areas. Because of soil physical and chemical effect, the rice yields are low, which is consistent with Srisomkiew et al. (2020) who reported that most of the cultivated areas are of low fertility.

Furthermore, three patterns were explained as follows, *Conventional jasmine rice production* is an approach to produce jasmine rice in this area. Combustion is chosen to eliminate jasmine rice straw after harvesting. To prepare the soil, generally, tractors are used to plow soil for helping the soil ventilate, weeding, and finally growing rice. Chemical fertilizers that include nitrogen, phosphorus, and potassium formula are used two times during the rainy season around May to June and August every year. Pesticides and herbicides are also used at least in the planting stage to protect the crop. It normally takes around four to five months to harvest jasmine rice that is based on climate. Harvested jasmine rice is then sent to rice storages and rice milling. *Organic jasmine rice production* is an optional approach to produce jasmine rice in this area. This approach is similar to conventional rice production but different in the detail. Plowing and rice residue fermentation is chosen to rice straw management for maintaining and increasing the organic matter in the soil. Organic fertilizer, manure, or plant fertilizer are used to prepare the soil. Bio fermentation of hormone is selected to help for increasing the yield. *Good Agricultural Practices jasmine rice production* is an alternative approach that was mixed between chemical and organic jasmine rice production. Residue fermentation is chosen to rice straw management in the post-harvest stage. Chemical and organic fertilizers are used to increase plant nutrient. Pesticides and herbicides are also used to protect the crop that was based on safe food production. Table 4 depicts the physical and chemical properties of soil in TKRH.

**Table 4:** Physical and chemical properties of soil in TKRH, Thailand

	Physical and chemical properties				
	pH	OM (%)	TN (%)	TP (mg-P/kg)	TK (mg-P/kg)
Roi- Et	4–6	0.80–2.70	0.04–0.09	9.00–28.00	46.00–57.00
Maha Sarakham	4–5	0.80–2.50	0.05–0.10	8.00–26.00	32.00–57.00
Surin	5–6	0.90–2.80	0.07–0.11	8.00–26.50	39.00–57.00
Si Saket	4–6	0.80–2.20	0.07–0.09	9.00–23.00	37.00–54.00
Yasothon	4–6	0.80–1.40	0.04–0.07	19.00–24.00	39.00–56.00

*OM* = Organic Matter; *TN* = Total Nitrogen; *TP* = Total Phosphorus; *TK* = Total Potassium

Table 4 describes that physical and chemical properties of soil in these areas were not suitable for increasing the rice yields. The soil pH was very strong acid to—slightly acidic. Soil organic matter found in the soil of Yasothon Province was low to slightly low; meanwhile, the other provinces were low to slightly high. Total nitrogen, phosphorus, and potassium remained at low to moderate levels. These findings are consistent with Saetung and Trelo-ges (2017b) who confirmed that the range of soil pH was 4.90—5.00. Moreover, Saetung and Trelo-ges (2017a) stated that soils fertility in TKRH area decreases from soil management after post-harvest and fertilizer use.

**Fig. 4:**
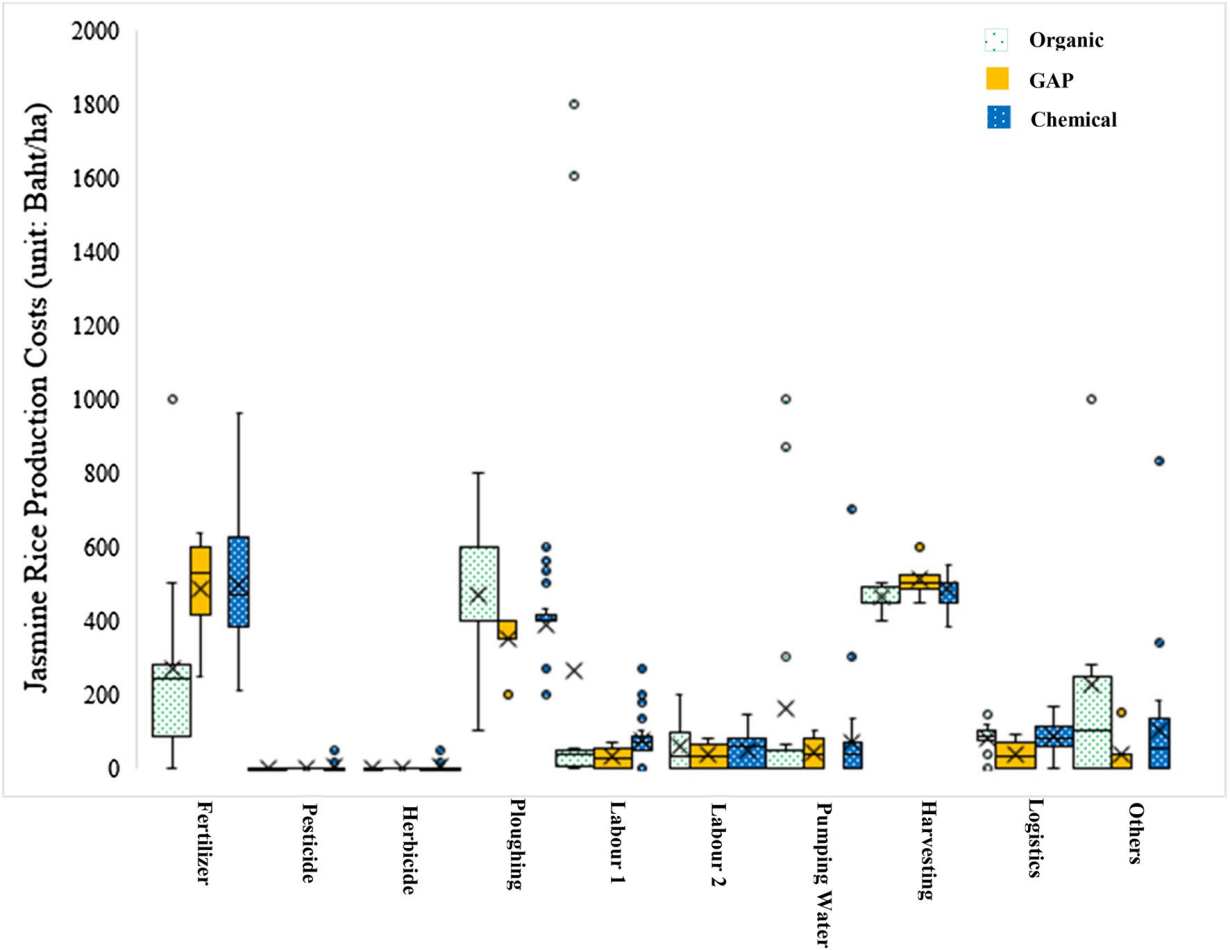
The comparison of jasmine rice production costs between Organic, GAP and chemical (unit: Baht/ha)

From interviewing we found that the amount of fertilizers used in chemical rice production was approximately 5.72 kg/ha (2.80 to 9.60 kg/ha). Fertilizer behavior of farmer is at least 1 to 2 times/crop cultivated that is based on budget, but it is not based on nutrients requirement of jasmine rice. At the same time, climate changes affect all precipitation quantities. Hence, the use of chemical fertilizer was reduced by 50 percent in 2019. This is consistent with the findings of Thai Central Chemical Public Company Limited (2019) who reported that insufficiency of rain during the annual rice cultivating period in the northeast region of Thailand has decreased the demand of chemical fertilizers. The limitation of fertilizer usage is rainfed because farmers will put it into the cultivated areas when it still has some water. On the other hand, the amount of fertilizers used in organic rice production was approximately 6.58 kg/ha (0.00 to 16.00 kg/ha). The ratio of organic fertilizer production consists of manure 1000 kg, phosphate rock 25 kg, rice bran 2 kg, and water approximately 50% humidity. Then, it is left for 24 days for fermentation. One of the important things that help to decrease chemical fertilizer usage is high cost as shown the data in Table 5 and Fig. 4.

**Table 5:** Organic, good agricultural practices, and chemical jasmine rice production costs unit: Baht/ha

	Organic rice producers; O (*n* = 14)		Good agricultural practices; G rice producers (*n* = 4)		Chemical rice producers; C (*n* = 31)	
	$\stackrel{-}{x}$	min–max	$\stackrel{-}{x}$	min–max	$\stackrel{-}{x}$	min–max
Fertilizer; Fer	271.60	0.00–100.00	484.79	250.00–637.50	497.24	210.00–960.00
Pesticide; Pet	0.00	0.00	0.00	0.00	2.17	0.00–50.00
Herbicide; Her	0.61	0.00–8.49	0.00	0.00	5.87	0.00–126.67
Plowing; MC1	478.57	100.00–800.00	312.50	200.00–400.00	385.73	200.00–600.00
Labor 1; MC2	269.43	0.00–1800.00	18.13	0.00–50.00	77.13	0.00–266.67
Labor 2; MC3	51.90	0.00–200.00	58.13	0.00–87.50	49.96	0.00–146.67
Pumping water; MC4	166.65	0.00–1000.00	18.75	0.00–75.00	68.54	0.00–700.00
Harvesting; MC5	462.27	450.00–500.00	5112.50	450.00–600.00	483.48	382.35–550.00
Logistics; Logis	71.24	0.00–120.00	70.83	0.00–145.83	83.61	0.00–166.67
Others	166.08	0.00–1000.00	250.00	0.00–1000.00	101.93	0.00–833.33

**Fig. 5:**
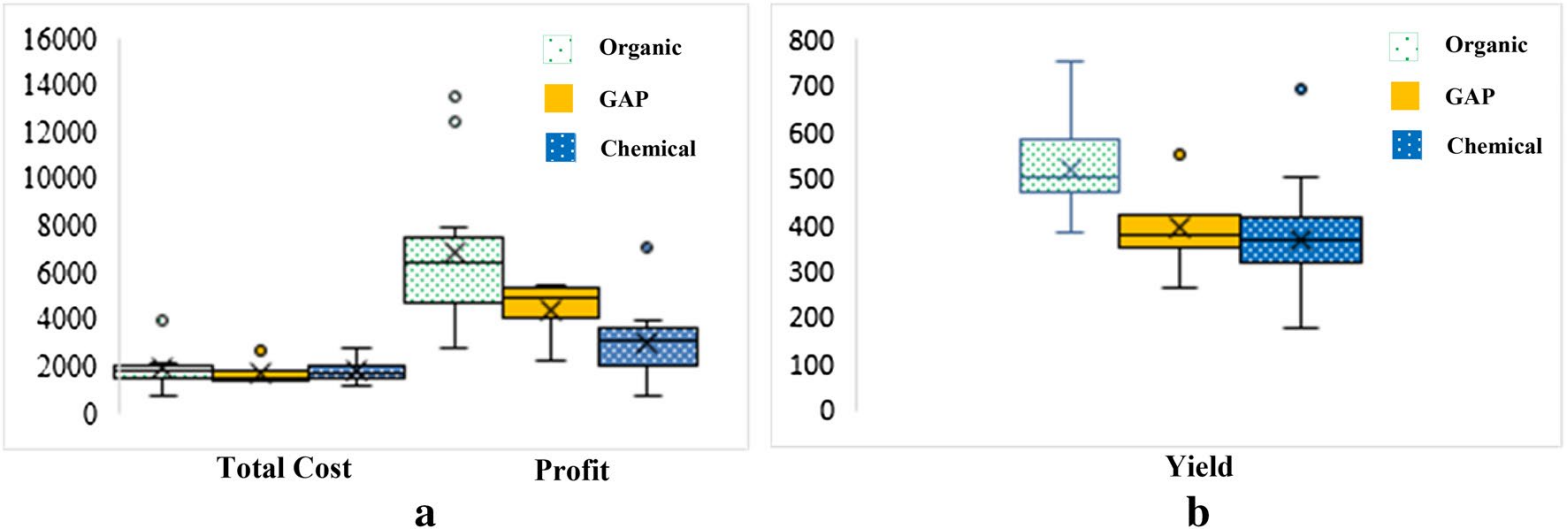
**a** cost and profit of three patterns of jasmine rice production, **b** yield of jasmine rice production

The total cost of chemical jasmine rice production was 1160.00 to 2733.33 baht/ha. At the same time, the other one was 730.00 to 3956.60 baht/ha. Management costs that includes plowing, labor, pumping water, and harvesting were the most transaction costs of rice production. Chemical rice production was 720.00 to 1850.00 baht/ha of management costs, but organic production was 530 to 3850 baht/ha of management costs. To conclude, chemical rice production costs were higher than the organic rice production costs as shown in Fig. 5(a). This is in line with Shukla et al. (2016); Tashi and Wangchuk (2016) who reported that chemical rice production costs were significantly higher than organic rice, as the variable input costs were significantly higher in the chemical rice production. In terms of profits, organic jasmine rice production was higher than chemical jasmine rice production. This was equal to 2813.75 to 13,480.00 baht/ha of profit; meanwhile, chemical jasmine rice production was equal to 1114.29 to 7,102.00 baht/ha of profit (Fig. 5(a)).

Furthermore, organic jasmine rice production yields were much higher than the chemical jasmine rice production. The highest yield of organic jasmine rice production was equal to 750.00 kg/ha. The chemical jasmine rice production yield was equal to 690.00 kg/ha. On the other hand, the lowest yield of organic jasmine rice production was equal to 380.00 kg/ha. Meanwhile, chemical jasmine rice production yield was equal to 177.78 kg/ha as shown in Fig. 5(b). One of the interviewees pointed out “*the difference between chemical and organic jasmine rice is the weight of jasmine rice. Organic jasmine rice is heavier than chemical. Most of the chemical jasmine rice is tiny and fine. Even though the organic rice yield is less about 100–200 kg, but the production costs are low and it fetches high prices, Hence, I changed my mind to follow organic rice production” (O12, interview)*. This was also echoed with other farmer who stated *“I change to do organic jasmine rice because the fertilizer prices are high, especially ureas which is approximately 800 to 1,000 Baht/ 50 kg. On the other hand, organic fertilizer was just 250 Bath/ 50 kg.”(O19, interview)* Furthermore, this study found that organic jasmine rice seeding production has a high net profit as seen in the interviews as one of the interviewees points out *“the prices of organic jasmine rice seeding was 29 Baht/kg (including market prices* + *Perseverance 5 Baht* + *Organic production 2 Baht). Meanwhile, chemical production and GAP system was approximately 18 to 20 bath/kg” (O15 and G16, interviews)*. To sum up, jasmine rice production in rainfed areas leads to high income and is suitable for production in such climate condition, whereas chemical jasmine rice production has a high production cost, has a low carrying capacity and more prone to plant disease.

This study found that there were two key challenges for jasmine rice production in TKRH. First, the topography, the cultivated area is based on the rainfed. Hence, it is at risk of drought, causing farmers unable to determine the time of planting. These areas have sandy or sandy loam soils that are unable to absorb water. Moreover, there are also saline or acidic soils in some areas and lack fertility and organic matter, resulting in low productivity while the cost of production is high. Second, in terms of human actions, lack of labor, high labor costs, and efficiency of jasmine rice production were an important part to produce for sustainable agriculture.

### Hotspots and environmental impacts of jasmine rice production

**Fig. 6:**
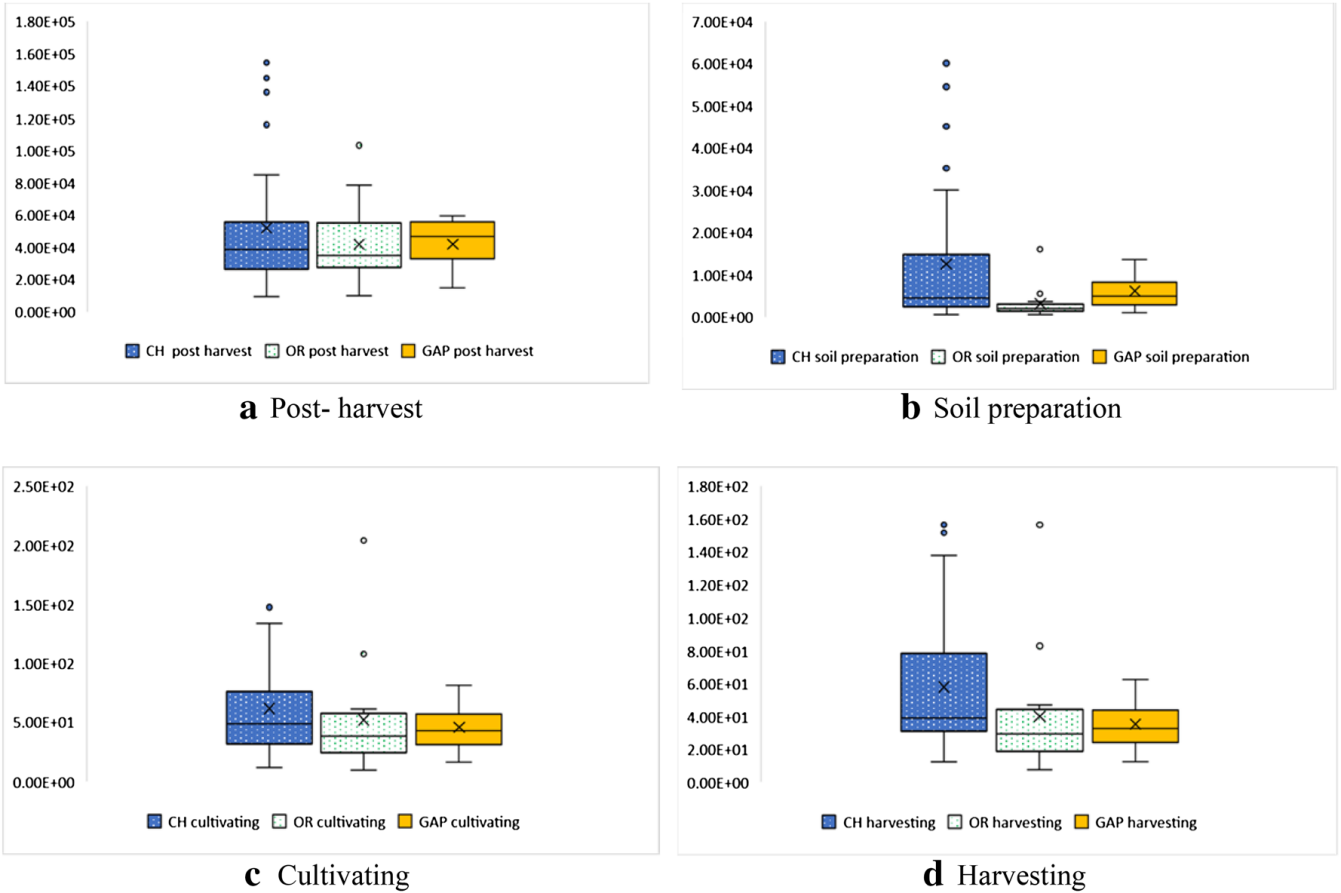
The GHG emissions of the process of three jasmine rice production approaches

The greenhouse gas (GHG) emissions of three jasmine rice production approaches consist of four steps. The result shows that most of the GHG emissions in rice production of all were the post-harvest management. Generally, straw residue combustion selected in chemical jasmine rice production was between 9.68E + 03 to 1.55E + 05 kgCO_2_eq of chemical paddy jasmine rice (Fig. 6(a)), consistent with the findings of Arunrat et al. (2016) who stated that burning of rice residue stages was the major source of GHG emissions. At the same time, straw residue fermentation was chosen in good agricultural practices (GAP) and organic jasmine rice productions, consistent with Jianyi et al. (2015) who reported anaerobic fermentation as one of the emission sources. These results of organic and GAP jasmine rice production were between 1.02E + 04 to 1.03E + 05 kgCO_2_eq of organic paddy jasmine rice and 1.47E + 04 to 5.95E + 04 kgCO_2_eq of GAP paddy jasmine rice as shown in Fig. 6 (b), (c), and (d). The GHG emissions of the process of three jasmine rice production approaches were slightly different. For jasmine rice production in this area, it was not necessary to hurry up planting the next crop, so post-harvest management should be considered as the effectiveness of rice cultivation. Yodkhum et al. (2017) demonstrated that organic fertilizers usage and crop residue fermentation results in the decrease of the GHG emissions when compared with rice straw combustion in chemical jasmine rice production.

Besides, the finding of this study explains the global warming potential (GWP)100, eutrophication, and acidification impact on emissions as follows:

#### Global warming potential (GWP)100

**Fig. 7:**
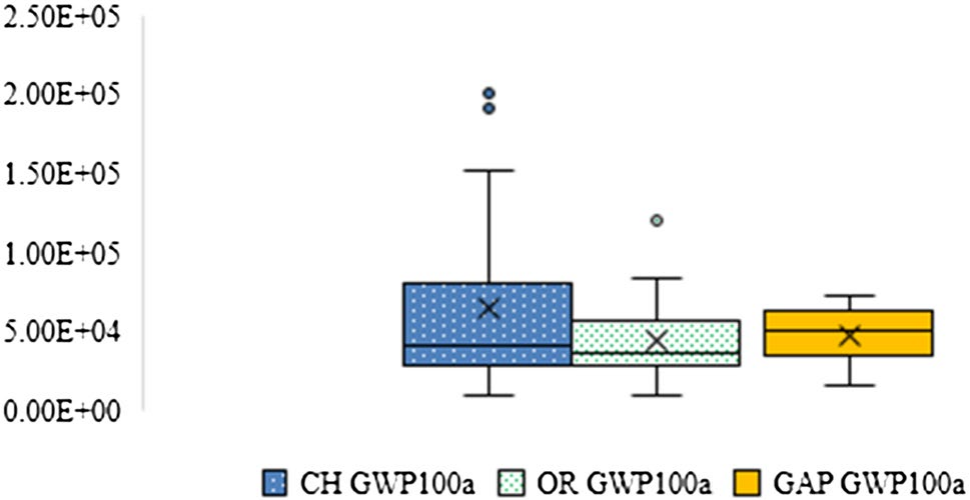
The GWP100 of chemical, organic, and GAP jasmine rice production

This study found that the GWP100 of chemical jasmine rice production was higher than GAP and organic jasmine rice production as shown in Fig. 7. It was between 1.03E + 04 to 2.06E + 05 kgCO_2_eq of GWP100. seventy-five percent of the GWP100 of post-harvest management from chemical jasmine rice production was especially from rice straw burning (9.68E + 03 to 1.55E + 05 kgCO_2_eq of GWP100) that causes an increase in the GHGs emissions. On the other hand, the GWP100 of GAP and organic jasmine rice production were between 1.57E + 04 to 7.34E + 04 and 1.07E + 04 to 1.20E + 05 kgCO_2_eq of GWP100, respectively. The high GHG emissions were released by rice straw fermentation from the post-harvest stage of both productions. The GWP100 of GAP productions was approximately 81%. At the same time, the GWP100 of organic productions was approximately 94%. Thus, GWP100 is one of the climate impacts on rice yields (Prabnakorn et al. 2018).

#### Eutrophication

**Fig. 8:**
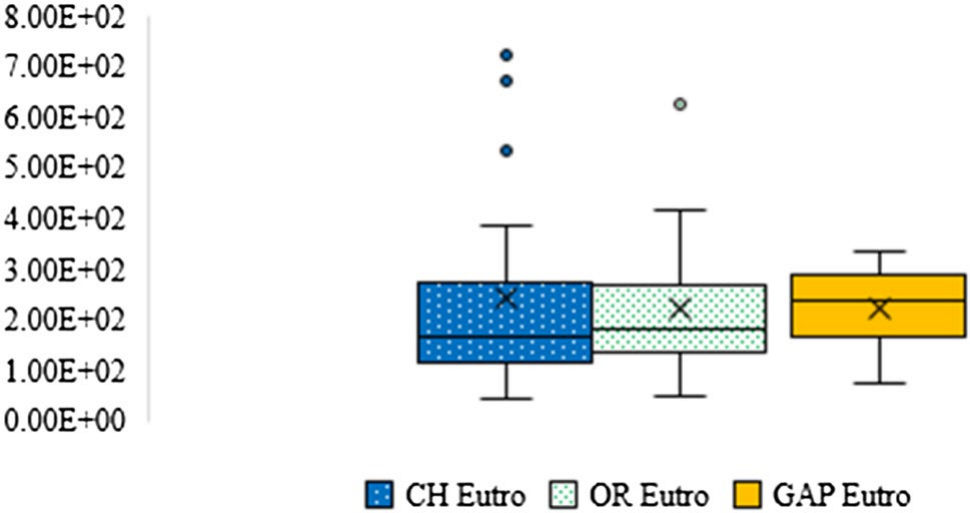
The eutrophication impact of chemical, organic, and GAP jasmine rice production

This study found that the impact of eutrophication of chemical jasmine rice production was higher than GAP and organic jasmine rice production as shown in Fig. 8. It was between 4.14E + 01 and 7.31E + 02 kgPO_4_-Eq. Eighty-one percent of the impacts were made from post-harvest management due to rice straw burning (4.01E + 01 to 6.42E + 02 kgPO_4_-eq) causing an increase in the GHG emissions. On the other hand, the impacts of GAP and organic jasmine rice production were between 7.50E + 01 to 3.35E + 02 and 5.08E + 01 to 6.27E + 02 kgPO_4_-eq, respectively. The high emissions were released by rice straw fermentation from the post-harvest stage of both productions. The impacts of GAP and organic productions were approximately 81%.

#### Acidification

**Fig. 9:**
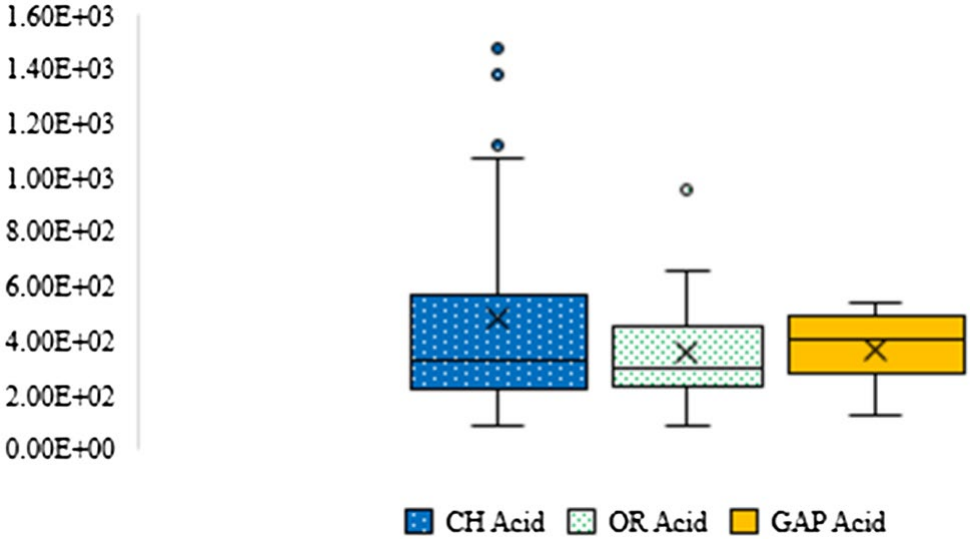
The acidification impact of chemical, organic, and GAP jasmine rice production

This study found that the impact of acidification of chemical jasmine rice production was higher than GAP and organic jasmine rice production as seen in Fig. 9. It was between 8.13E + 01 to 1.48E + 03 kgSO_2_eq. Eighty-three percent of the impacts were made from post-harvest management that was due to rice straw burning (7.79E + 01 to 1.25E + 03 kgSO_2_eq) and causes an increase in the GHG emissions. On the other hand, the impacts of GAP and organic jasmine rice production were between 1.22E + 02 to 5.41E + 02 and 8.37E + 01 to 9.53E + 02 kgSO_2_eq, respectively. The high emissions were released by rice straw fermentation from the post-harvest stage of both productions. The impacts of GAP production were approximately 88%. At the same time, the impacts of organic production were approximately 96%.

The results of this study suggest that organic jasmine rice production is the most suitable rice production. The area context is based on topography that sandy soil is still required to increase the organic matter into the soil for enhancing the capacity of the nutrient available for the plant. In terms of climate, this alternative approach was low in emission impact, consistent with Arunrat et al. (2020) who reported that the GHG emissions can be reduced in the rice cultivation through soil organic carbon (SOC) improvement. This confirmed the finding of Tricase et al. (2018) who found that the organic cultivation is the most environmentally sustainable solution. Moreover, these results were supported by Tellez-Rio et al. (2017) who note that crop rotation was a good agricultural approach to control between GHGs emission, GWP, yield-scaled N_2_O emissions, and N surpluses. Although Arunrat et al. (2018) who reported that temperature has risen and will slightly decrease rice yield, however, the most important thing for increasing the rice yields was enhancing nitrogen availability to the soil from rice straw fermentation. Furthermore, rice straw burning selected in post-harvest management has high emission impact and ruins the soil structure in low-fertility soils. Meanwhile, Chen et al. (2011) reported that GAP system management can increase yields but also requires increasing usage of fertilizers.

## Conclusions

The findings of this study show that the alternative approach that is suitable for jasmine rice production in these areas is organic jasmine rice production. Rice straw is important and must concentrate on jasmine rice production. Good crop productivity relies on good soil structure, especially the soil preparation stage should concentrate to control the jasmine rice yields and environmental emissions. In terms of increasing the jasmine rice yields, it is affected by rice straw that is returned to the soil. This helps to improve the soil structure for fertilizer absorption. At the same time, production costs and environmental emission were decreased by this procedure. On the other hand, stubble burning should be avoided because of their effect on the destruction of soil and the high greenhouse gas emission impacts. Thus, our findings add valuable insights to reduce environmental impacts.

Following the eco-efficiency of the World Business Council for Sustainable Development (WBCSD), this approach aims to explain the alternative way for sustainable agricultural development. There are three important issues. First, *Reducing the consumption of resources*- rice straw was returned into the soil in the post-harvest stage of organic jasmine rice production. This was a positive step when compared with chemical jasmine rice production. Rice straw is important to improve the soil structure for increasing the carrying capacity of the nutrient plant needed. This management will also help to reduce fertilizer usage. Even though the efficiency of chemical fertilizer is higher than organic fertilizer, in the long run, it causes damage to the soil structure and the soil cannot absorb the nutrient released. Second, *Reducing the impact on nature- *result from this study illustrated that the environmental impact of organic jasmine rice production was lower than good agricultural practices (GAP) and chemical jasmine rice production. Finally, *Increasing product or service value-* not only the rice production costs are low but also the jasmine rice yields are high. When calculating the eco-efficiency of jasmine rice production was equal to 0.02 to 0.40. Thus, this study provides a useful recommendation for policymakers to promote organic jasmine rice production as adaptive management for sustainable agriculture and future food security. Our findings can help the policymakers to design future ago-policy promoting organic farming practices to address the sustainability challenges. Our findings also recommend planning to determine the potential area for development as a cluster for safe food production in Thailand and beyond.

This study was focused on jasmine rice production that is based on rainfed. The results of this case study do not represent rice production that is based on irrigation. Hence, this is a limitation of this study. The results suggest that additional harvesting data should be collected to cover the overall rice production. The context of these areas supports the organic and GAP jasmine rice production, so future research must consider the context of the areas used.

### Contributions and future research

The finding of this study relates to the soil physical properties in the TKRH region as these areas have sandy or sandy loam soils. Therefore, organic matter quantities in these areas are extremely low. The poor quality of the soil is unable to absorb water or fertilizers, and hence, conventional yield is low in this region. The key fact that helps to increase the yields of organic production is organic matter quantities in the soil. The quantity of organic matter is based on post-harvest and soil preparation steps. Organic rice production opts for rice straw fermentation or plowing which helps to improve the organic matter in the soil thus improving the yield, whereas rice straw combustion is followed in conventional rice production which results in reduced organic matter in soil affecting the yield. Our results thus advocate organic rice production as a better alternative to conventional production where there is poor soil quality. Hence, the result of this study would help to support jasmine rice production approach that achieve the sustainability goals. However, the limitation of this study is based on rainfed, so the results of these would not represent jasmine rice production which is based on the irrigation. Future studies should study the another one for assessing the environmental impacts of jasmine rice production. Furthermore, future studies should also focus the other impact categories (such as fine particulate matter and human toxicity) to understand the full impact of rice production on the environment.
